# The Immunomodulatory Effects of Plant Extracts and Plant Secondary Metabolites on Chronic Neuroinflammation and Cognitive Aging: A Mechanistic and Empirical Review

**DOI:** 10.3389/fphar.2017.00117

**Published:** 2017-03-10

**Authors:** Christina Kure, Jorinde Timmer, Con Stough

**Affiliations:** Swinburne Centre for Human Psychopharmacology, Swinburne UniversityHawthorn, VIC, Australia

**Keywords:** neuroinflammation, cognitive aging, plant based extracts, herbal medicine, antioxidants, prakriti, Ayurveda, personalized herbal medicine

## Abstract

Advances in healthcare have considerably improved the life expectancy of the human population over the last century and this has brought about new challenges. As we live longer the capacity for cognitive aging increases. Consequently, it has been noted that decline in cognitive performance in the elderly in domains of reasoning, problem solving skills, attention, processing speed, working memory and episodic memory is a significant societal problem. Despite the enormity of this issue there are relatively few interventions for cognitive aging. This may be due to our current state of knowledge on biological factors that underpin cognitive aging. One of the biological contributors to cognitive aging is chronic neuroinflammation. This review will provide an overview of the peripheral and central mechanisms involved in chronic neuroinflammation and how neuroinflammation may be related to age-associated cognitive decline. Plant based extracts including herbal and nutritional supplements with anti-inflammatory properties will be examined in relation to their utility in treating age-related cognitive decline. Plant based extracts in particular offer interesting pharmacological properties that may be quickly utilized to prevent cognitive aging.

## Introduction

Advancements in healthcare have improved our life expectancy over the last century (United Nations DoEaSA, 2013) and as we live longer the capacity for cognitive aging increases (Bishop et al., 2010). A decline in cognitive performance in older people in domains of reasoning, problem solving skills, attention, processing speed, working memory and episodic memory is a significant societal problem (Simen et al., 2011). Therefore, interventions that improve cognitive function in older people are needed to reduce this burden on society. One biological mechanism related to cognitive aging is chronic neuroinflammation (Simen et al., 2011; Lim et al., 2013; Patterson, 2015; Wu et al., 2016). Acute inflammation consists of local and systemic interactions of several cell types, chemical signals and signaling pathways comprising the innate and the adaptive immune system. While acute neuroinflammation has an important role in maintaining neural homeostasis, protecting the brain from disease, and removing damaged tissue and repairing injury; chronic low-grade neuroinflammation is destructive (Medzhitov, 2008). Associated with aging, chronic neuroinflammation is a result of a deregulated acute phase response of the innate immune system effecting surrounding neural tissue on a molecular, structural and functional level (Nguyen et al., 2002).

## Plant extracts and plant secondary metabolites that target neuroinflammation and may improve cognition

The physiological mechanism for cognitive impairment is not refined to one biomarker or area of the body. It is complex involving multiple mechanisms including overexpression of peripheral and neural inflammatory processes, BBB leakage, and over activation of microglia and astrocytes. The process of drug discovery to prevent cognitive decline over an adult's life needs to therefore explore substances that target these mechanisms that are related to cognitive aging. Pharmaceutical interventions, possibly because cognitive aging is not currently defined as a medical illness, have been poor. Therefore, we need to turn our attention to other pharmacologically active substances that may reduce chronic neuroinflammation and improve cognitive function in older people. Plant based extracts possess multiple pharmacological actions on the human brain and therefore may give rise to multiple therapeutic uses within this domain.

In the first part of this review we outline the peripheral and central chronic inflammatory processes that have a complicated relationship with age-related cognitive decline. In the second part of this review we outline key plant extracts and plant secondary metabolites that may address the neuroinflammatory mechanism associated with age-related cognitive changes.

PART I: AGE-RELATED PERIPHERAL AND CENTRAL CHRONIC NEUROINFLAMMATORY PROCESSES AND COGNITIVE PERFORMANCE.

## Age-related alterations of pro- and anti-inflammatory cytokine levels

Cytokines are a class of small proteins, divided into pro- and anti-inflammatory cytokines, which are secreted by several macrophages including microglial cells and astrocytes in the brain (Cohen and Cohen, 1996). Cytokines are primary messengers important in mediating neuroinflammation. They activate a range of immune cells and promote the production of inflammatory mediators including chemokines and acute phase proteins (Holdsworth and Gan, 2015). Non-pathological aging is often associated with an increased inflammatory profile reflected in elevated levels of circulatory pro-inflammatory mediators. Elevated blood levels of the pro-inflammatory cytokine interleukin (IL)-6 (Wei et al., 1992; Roubenoff et al., 1998; Forsey et al., 2003; Stowe et al., 2010; Álvarez-Rodríguez et al., 2012), IL-1β and tumor necrosis factor-alpha (TNF-α) (Álvarez-Rodríguez et al., 2012), and the acute phase c-reactive protein (CRP; Roubenoff et al., 1998) have been reported in healthy elderly people. The excessive production of cytokines may have adverse consequences, with TNF-α for instance inducing demyelination and axonal degeneration (Stoll et al., 1993).

Earlier studies have observed changes in the pro-inflammatory to anti-inflammatory cytokine ratio during aging (Forsey et al., 2003). As opposed to an increase in pro-inflammatory cytokine levels, some researchers failed to observe an increase in anti-inflammatory cytokines (e.g., IL-10) with increased age (Forsey et al., 2003; Stowe et al., 2010). Conversely, Álvarez-Rodríguez and colleagues did not observe a change in cytokine ratio, but rather an increased overall inflammatory profile including increased levels of IL-10 (Álvarez-Rodríguez et al., 2012). A possible explanation for these conflicting results could be that cytokine profiles differ not only with age, but also according to ethnicity (Stowe et al., 2010). Additionally, the use of different methods across studies to assess circulatory inflammatory marker levels and assay sensitivity could also explain the contradictory findings between studies.

Peripheral cytokines have the ability to communicate with the central nervous system (CNS) via: (United Nations DoEaSA, 2013) receptors on endothelial cells of the blood brain barrier (BBB); (Bishop et al., 2010) active transport across tight junctions of the BBB; and (Simen et al., 2011) the vagal nerve (Maier et al., 1998; Banks, 2005; Erickson et al., 2012). In the CNS, cytokines are primary messengers important in mediating neuroinflammation. They activate a range of immune cells and promote the production of inflammatory markers including chemokines and acute phase proteins (Holdsworth and Gan, 2015).

## Age-related increased blood brain barrier permeability

The blood brain barrier (BBB) is a dynamic interface between the peripheral circulation and the brain parenchyma consisting of endothelial cells lining the brain capillaries. The BBB tightly regulates the transport of blood-derived molecules, proteins and cells, inflammatory cytokines, into or out of the CNS via tight junctions involving active transport or receptor binding (Abbott and Friedman, 2012; Erickson et al., 2012). Additionally, the BBB maintains a complex relation with several cell types located in the brain parenchyma, such as astrocytes and microglial cells, and as such plays a key role in the communication between the CNS and the immune system (Erickson et al., 2012; Abbott and Friedman, 2012).

Animal and clinical studies have shown an age-related increase of BBB permeability in healthy individuals (Toornvliet et al., 2006; Farrall and Wardlaw, 2009; Blau et al., 2012). Recently, advanced magnetic resonance imaging (MRI) techniques have shown increased BBB permeability in healthy older people without cognitive impairment aged between 55 and 90 years in specific areas of the brain important for cognitive functioning, especially in the hippocampus (Montagne et al., 2015). Various factors are believed to effect BBB integrity. In animal studies, Elahy et al. (2015) provided evidence for inflammation driven BBB dysfunction and decreased tight junctions in aged mice compared to young mice (Elahy et al., 2015). Furthermore, recent evidence obtained from experiments on animal models suggests that a lack of normal gut microbiota in germ-free mice is another potential regulator of BBB integrity (Braniste et al., 2014). However, the mechanism for this gut-brain relationship in modulating BBB integrity is unknown (Braniste et al., 2014).

## Age-related changes in glial cell activation: microglial cells and astrocytes

The brain parenchyma consists of neurons and glial cells. Glial cells are divided into macro glial cells, as astrocytes, and microglial cells. Microglial cells are the resident macrophages and main orchestrators of the immune response in the CNS. A key role of the microglial cells is to respond to signals from the peripheral immune system and observed pathogens or injury and neuronal signals in the CNS (Kreutzberg, 1996; Kierdorf and Prinz, 2015). At rest and under normal homeostatic circumstances, microglial cells express a branched morphology and ceaselessly observe their microenvironment for damaged tissue or pathogens (Nimmerjahn et al., 2005; Rivest, 2009; Torres-Platas et al., 2014). Upon activation, microglial cells undergo morphological and functional transformations: they retract their branches and form hypertrophic cell bodies and express immune mediators as pro-inflammatory and anti-inflammatory cytokines (Hanisch, 2002; Torres-Platas et al., 2014). After homeostatic balance has been restored microglial cells return to their “observing” phenotype and transform back to their resting state morphology (Nimmerjahn et al., 2005; Rivest, 2009; Karperien et al., 2013). Astrocytes on the other hand are the main glial cells of the brain parenchyma and maintain a close relationship with the BBB permeability rate forming the glial limitans of the BBB (Abbott et al., 2006; Sofroniew, 2015). Astrocytes have been shown to modulate microglial cell activity (Von Bernhardi and Eugenín, 2004; Tichauer et al., 2007), perform an important role in synaptic transmission in the synaptic cleft (Shigetomi et al., 2008; Perea et al., 2009) and influence synaptic function (Ota et al., 2013).

Normal aging is associated with glial senescence characterized by a primed, activated state of the microglial cells and astrocytes. In animal models microglial cells of aged individuals have an activated morphology, expressing hypertrophic cell bodies in the absence of disease (Hwang et al., 2008). In addition, they express increased mRNA for pro- and anti-inflammatory cytokines and other biomarkers, such as, IL-1β, IL-6, TNF-α, IL-10, and major histocompatibility complex class II proteins (Sheffield and Berman, 1998; Sierra et al., 2007; Henry et al., 2009; Wu et al., 2016). This is consistent with the findings of an overall increased inflammatory cytokine profile in healthy elderly people (Álvarez-Rodríguez et al., 2012). However, a recent PET study comparing healthy elderly to young people concluded that aging was not associated with increased activity of microglial cells, but rather degeneration of microglial cells (Suridjan et al., 2014).

In the prefrontal cortex and areas of the hippocampus of aged rats an increased number of astrocytes with an altered morphology have been assessed and these astrocytes are larger in size, indicating an activated profile (Amenta et al., 1998). In addition, astrocytes in the aging brain of animals and humans express an activated phenotype reflected by an increased production of the astrocytic glial fibrillary acidic protein (GFAP) (Nichols et al., 1993; David et al., 1997). The age-related increases in GFAP has a similar profile as activated astrocytes during acute inflammation (Pekny and Pekna, 2004).

## Oxidative stress and chronic neuroinflammation

In the brain parenchyma, microglial cells, together with intracellular mitochondria, are the main producers of reactive oxygen species (ROS), such as nitric oxide. ROS are a natural by-product of energy production (Gemma et al., 2007). To maintain a homeostatic balance microglial cells are involved in antioxidant defense mechanisms and during inflammation microglial cells are also the main producers of antioxidants in the CNS, such as glutathione (Hirrlinger et al., 2000; Dringen, 2005). A homeostatic balance between these two processes is of importance for adequate energy production of microglial cells (Gemma et al., 2007). Both underproduction as well as overproduction of ROS result in dysfunctional cells and intercellular communication (Gemma et al., 2007).

Animal evidence shows an age-related overproduction of ROS in primed microglial cells (Hayashi et al., 2008; Nakanishi and Wu, 2009). During age-related chronic inflammation, it is hypothesized that antioxidants are depleted and an overproduction of ROS occurs. This results in an imbalance between ROS and antioxidants, causing oxidative stress (Gemma et al., 2007; Njie et al., 2012). Indeed, oxidative stress caused by depletion of antioxidants has shown to activate inflammatory pathways, such as Nuclear Factor kappa B (NFκB) in an experimental animal model (Lee et al., 2010). As a result, microglial cells and astrocytes were shown to be activated resulting in elevated amounts of IL-6 and TNF-α (Lee et al., 2010; Njie et al., 2012).

## Chronic stress and neuroinflammation

On presentation of an internal or external stressor a series of (neuro) endocrine reactions take place. The neuroendocrine cascade is initiated via the hypothalamic pituitary adrenal (HPA) axis and results in the excretion of several neuropeptides and stimulating factors and the eventual release of glucocorticoids (Aguilera, 2011). Glucocorticoids modulate HPA-axis activity via feedback to the pituitary, hippocampus and hypothalamus (Aguilera, 2011). Acute stress responses are essential for survival and homeostatic rebalance and have an immunosuppressive effect (Aguilera, 2011; Tian et al., 2014; Duque Ede and Munhoz, 2016). However, chronic exposure to stress hormones may predispose to immune alterations resulting in an increased inflammatory response in the brain via activation of the NF-κB pathway and increased release of pro-inflammatory cytokines (Munhoz et al., 2006; Aguilera, 2011; Tian et al., 2014; Duque Ede and Munhoz, 2016).

Several animal studies have shown that the prefrontal cortex and the hippocampus, areas with high density of glucocorticoid receptors, are sensitive to the neuroinflammatory effects of glucocorticoids and a range of pro-inflammatory responses are induced via increased microglial activity, activation of the NF-κB pathway and TNF-α and IL1β expression (de Pablos et al., 2006; Munhoz et al., 2006, 2010). In addition, Schiavone et al. (2016) demonstrated that in rats isolation-induced chronic stress increased BBB permeability, reflected by increased expression of matrix metalloproteinases and increased expression of IL-6 (Schiavone et al., 2016).

A longitudinal study demonstrated that chronic stress resulted in an age-related increased expression of IL-6 (Kiecolt-Glaser et al., 2003). However, another study found no age-related correlation between life event stress and memory functioning in adults (Korten et al., 2014). In an animal model though, McKim et al. (2016) showed that chronic stress induced hippocampal inflammatory responses characterized by increased pro-inflammatory cytokine expression and microglial activation. This also resulted in transient spatial memory impairments (McKim et al., 2016).

## Hippocampal neurogenesis and neuroinflammation

Hippocampal neurogenesis plays an important role in memory consolidation and spatial learning, processes in which the hippocampus exerts a key role (Ojo et al., 2015). Hippocampal neurogenesis has shown to diminish in aged animals (Ojo et al., 2015). In human *post-mortem* studies a moderate decline in hippocampal neurogenesis has been observed (Knoth et al., 2010). Increased neuroinflammation and especially an increased activation of microglial cells is thought to underlie this diminished hippocampal neurogenesis (Ojo et al., 2015). Indeed, Ekdahl and colleagues showed that increased microglial activity inhibited the formation of new neurons in the hippocampal dentate gyrus (Ekdahl et al., 2003). However, a human *post-mortem* study in patients with Alzheimer's disease showed increased hippocampal neurogenesis and the findings of an *in vitro* study suggested that astrocyte excreted IL-6 promotes hippocampal neurogenesis (Jin et al., 2004; Oh et al., 2010). Although a study with mice showed age-related decline in neurogenesis which was correlated with cognitive decline, a study in Rhesus monkeys showed that a decrease in hippocampal neurogenesis is not highly related to age-related cognitive decline (Villeda et al., 2011; Ngwenya et al., 2015). Therefore, the exact relation between hippocampal neurogenesis, neuroinflammation and age-related cognitive decline should be investigated further.

## Chronic low-grade neuroinflammation and cognitive aging

A growing body of preclinical and clinical studies indicate that the age-related physiological and functional changes of the immune system are associated with age-related cognitive decline. Cytokines, microglial cells and astrocytes are involved in molecular mechanisms underlying cognitive functions, such as neurogenesis, synaptic transmission, synaptic pruning, long-term potentiation and synaptic plasticity (Newman, 2003; McAfoose and Baune, 2009; Morris et al., 2013; Ota et al., 2013). Moreover, the pro-inflammatory cytokines IL-1β, TNF-α and IL-6 are particularly overexpressed on microglial cells and astrocytes in areas of the hippocampus and prefrontal cortex (David et al., 1997; Liu et al., 2012).

Expression of pro-inflammatory cytokines in the brain above basal level has shown to impair synaptic plasticity and hippocampal-dependent memory learning in rodents (Sierra et al., 2007; Barrientos et al., 2010; Hein et al., 2010; Norden and Godbout, 2013). In coherence with these results, Blau et al. (2012) observed in an imaging study that compared to young rats increased BBB permeability in the perivascular space and hippocampal areas was associated with age-related dysfunction of long-term potentiation in the old rats, a process underlying the formation of memories (Blau et al., 2012). In addition, age-related dysfunction of long term potentation (LTP) through chronic systemic inflammation might be mainly caused by neuroinflammation induced by microglial cells (Liu et al., 2012).

**Figure 1:**
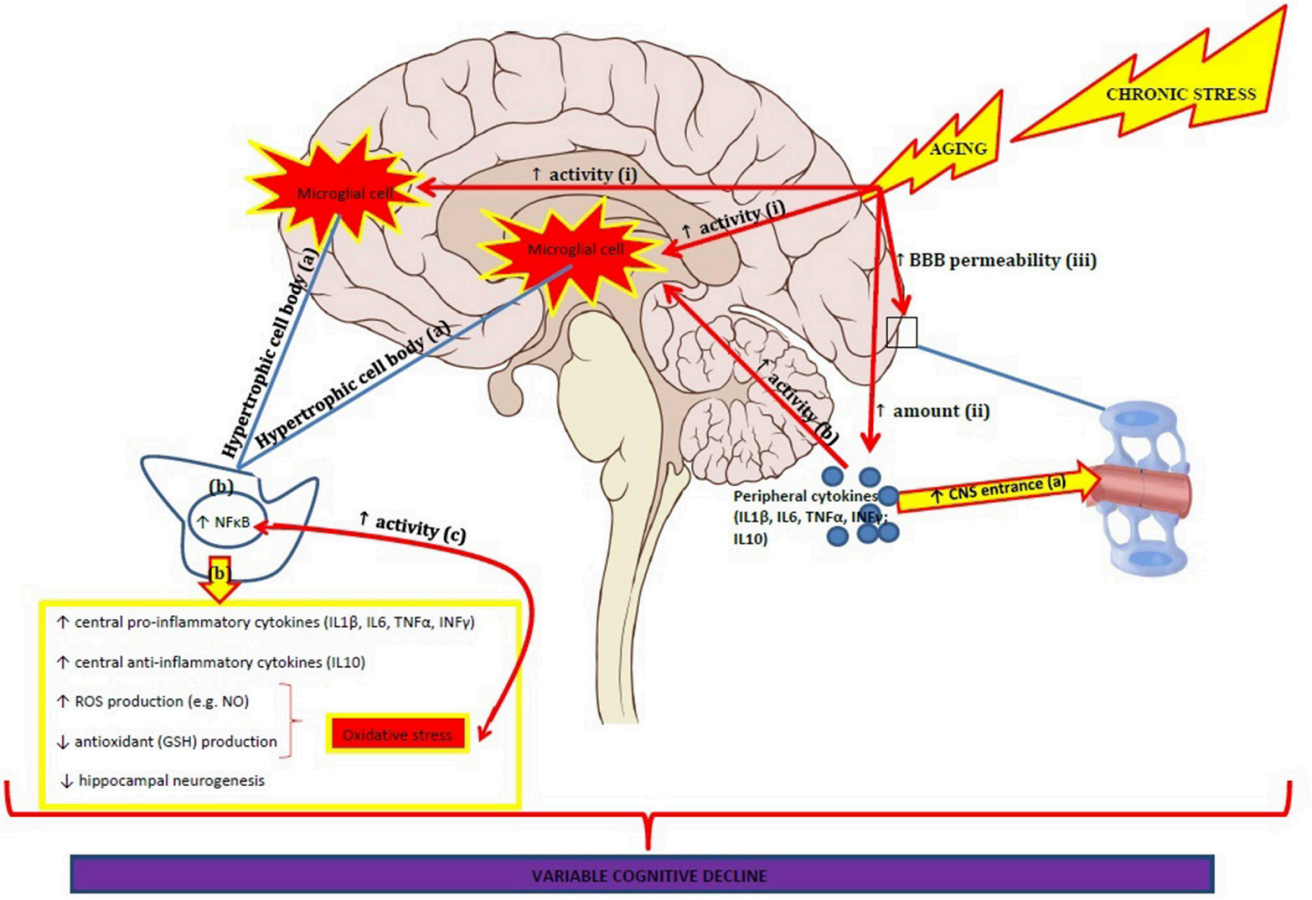
**Age-related effects on neuroinflammation are characterized by:**.
an increased activity of microglial cells, especially in the hippocampus and prefrontal cortex.this is characterized by microglial cells with hyperthrophic cell bodies.which is accompanied by increased NF-κB expression; increased pro-and anti-inflammatory cytokine production; increased ROS production and decreased antioxidant production which results in oxidative stress; decreased hippocampal neurogenesis.Oxidative stress and NF-κB increase each other.an increased amount of peripheral pro- and anti-inflammatory cytokines.(iii) increased BBB permeability.increased entrance of peripheral cytokines.peripheral cytokines induce increased activity of microglial cells. The age-related neuroinflammatory processes are amplified when exposed to chronic stress. The neuroinflammatory changes result in variable cognitive decline.

In healthy elderly people IL-6 has been negatively associated with encoding and recall of memories as well as with processing speed, executive functions and global cognitive functioning (Ravaglia et al., 2005; Elderkin-Thompson et al., 2012; Trollor et al., 2012). Another study found that increased levels of CRP were associated with poorer memory and smaller medial temporal lobe volumes in healthy elderly people (Bettcher et al., 2012). However, Palta et al. (2015) were unable to replicate these results in their longitudinal study with healthy elderly women. Neither IL-6 nor CRP levels were associated with immediate and delayed memory or executive functions, although IL-6 was negatively associated with processing speed (Palta et al., 2015). Comparing these studies is difficult with the latter study using different methodologies and analyses (i.e., dividing inflammatory markers into tertiles and measured non-fasting blood inflammatory levels). Diurnal rhythms of cytokines in plasma and serum have also shown to fluctuate with age (Altara et al., 2015). Interestingly, a recent study reported an increased oxidative stress status and lower antioxidants levels as well as an increased inflammatory profile in institutionalized healthy elderly people compared to non-institutionalized healthy elderly people and these variables correlated with lower cognitive performance with oxidative stress best predicting cognitive decline (Baierle et al., 2015). Together the results from these studies indicate that the underlying mechanisms involved in chronic neuroinflammation and oxidative stress negatively influence cognition across several domains as we age. Differences between studies might be due to a variety of psychological and cognitive test batteries used as well as different methodologies applied to measure inflammatory biomarkers (see Pase and Stough, 2013). Longitudinal intervention studies targeting neuroinflammation and oxidative stress for the elderly are urgently required. Figure 1 summarizes the potential inflammatory pathways that are involved in cognitive decline.

PART II: PLANT EXTRACTS AND PLANT SECONDARY METABOLITES THAT MAY ADDRESS THE NEUROINFLAMMATORY MECHANISMS ASSOCIATED WITH AGE-RELATED COGNITIVE.

## Polyphenols and alkaloids

### Resveratrol

Resveratrol is a plant-derived polyphenol found in red wine and grapes and has the capability to protect and cross the BBB. In their study, Turner et al. (2015) observed that resveratrol and its metabolites were present in plasma and the CNS and based on this observation suggest that penetration of these metabolites across the blood brain barrier occurred. As shown in mouse models resveratrol may protect the integrity of the BBB by preserving normal cerebral endothelial function and thus BBB permeability (Lin et al., 2010; Zhao et al., 2015). Resveratrol protects the BBB and also inhibits beta-amyloids accumulating in the hippocampus as demonstrated in AD rat models (Zhao et al., 2015). Resveratrol may also play a role in neuroprotection by inhibiting interleukin beta (IL-1β) and BV-2 murine microglial cells (Abraham and Johnson, 2009), and with its derivatives inhibiting microglial activation and reducing the production of pro-inflammatory factors (Candelario-Jalil et al., 2007; Meng et al., 2008). These mechanisms may explain the potential therapeutic effects of resveratrol on preventing cognitive decline as demonstrated in clinical trials. For example, a recent 26 week randomized clinical trial (RCT) with resveratrol supplementation (200 mg/d) in older overweight individuals resulted in improved retention of words and reduced body fat. MRI scans during cognitive tasks performance, have shown that compared to placebo, resveratrol increases cerebral blood flow in the prefrontal cortex as measured by changes in total hemoglobin concentrations (Kennedy et al., 2010). Other neuroimaging studies have also revealed increases in hippocampal functional connectivity, which correlated with improvements in retention scores and glycated hemoglobin, an indicator of improved glucose metabolism (Witte et al., 2014). Brain imaging also showed an increase in brain volume loss in individuals with mild to moderate AD who took a high-dose resveratrol intervention for 52-weeks (Turner et al., 2015).

### Curcumin

*Curcumin*, the yellow pigment of turmeric (*Curcuma lunga*) is a powerful antioxidant and consumption in the diet has been linked to reduced AD rates. The mechanism by which curcumin is neuroprotective may be by decreasing Aβ plaques, metal-chelation, and decrease microglia activation and protection of the BBB breakdown (Jiang et al., 2007). Jiang et al. (2007) studied the effects of curcumin injection in rats exposed to cerebral ischemia-reperfusion. They found that curcumin reduced infarct volume, neurological deficit and BBB permeability (Jiang et al., 2007). In other animal studies, curcumin was shown to inhibit free radicals (nitric oxide) and inflammatory markers (TNF-α, IL-1α, and IL-6) produced by microglia (Lee et al., 2007) and reduced levels of an astrocyte marker (GFAP) in animal models (Lim et al., 2001). Few randomized, double blind, placebo-controlled trials examining the effects of curcumin on cognitive function have been conducted in humans. Although curcumin was shown to decrease Aβ-plaque deposition, it does not seem to provide benefits on cognition in AD patients (Baum et al., 2008). Yet in healthy elderly adults acute doses (1 and 3 h post intervention) of a special curcumin extract (400 mg Longvida®) significantly improved performance on sustained attention and working memory compared to controls (Cox et al., 2015), although these findings were acute rather than chronic effects. A more limited range of effects were seen chronically after a 4 week administration period (Cox et al., 2015).

### Pinocembrin

Pinocembrin is one of the flavanones found in propolis and honey with actions useful in preserving cognitive function. Preclinical studies have shown pinocembrin treatment prevents or improves cognitive functioning (Guang and Du, 2006; Liu et al., 2014). The mechanisms by which procembrin improves cognition may be through its neuroprotective effects, reducing inflammatory mediators, reducing glial activation and reducing ROS production (Guang and Du, 2006; Liu et al., 2014). In a recent animal study, pinocembrin ameliorated cerebral damage caused by global cerebral ischemia-reperfusion. These neuroprotective effects (Lin et al., 2010) were attributed to pinocembrin suppressing damaging biomarkers including oxidative stress and inflammation (Saad et al., 2015). Another mechanism may be through improving brain edema, reduced BBB permeability and improve cerebral blood flow (Meng et al., 2011). Future clinical studies are required to explore the possible effects of pinocembrin on preventing cognitive function in humans.

### Epigallocatechin-3-gallate (EGCG)

Epigallocatechin-3-Gallate (EGCG) is a compound extracted from green tea (*Camellia sinensis*) that has polypharmacological actions contributing to its potential role in preventing cognitive decline. EGCG inhibits the production of Aβ-induced neuroinflammatory response of microglia (TNF-α, IL-1β, IL-6, and inducible nitric oxide synthase), protects against neurotoxicity and inhibits ROS (Cheng-Chung Wei et al., 2016). Similarly, in another study in animals with increased neuroinflammation and memory impairment, EGCG prevented the activation of astrocytes and increase in cytokines (TNF-α, IL-1β, IL-6) (Lee et al., 2013). Moreover, in a cerebral ischemia mouse model, Wu and colleagues (2012) found that chronic treatment with either a green tea extract or EGCG improved learning and memory deficits. Additionally, both treatments resulted in elevated levels of antioxidant levels and activity (Malondialdehyde, glutathione, and superoxide dismutase) in the cerebral cortex and hippocampus (Wu et al., 2012). In addition, EGCG had anti-inflammatory effects in microglia cells. These studies demonstrate that EGCG can prevent memory impairments by potentially inhibiting neuroinflammatory biomarkers and reducing oxidative stress. To date most of the research on EGCG has been preclinical. One pilot study demonstrated that a single dose of EGCG (135 mg) modulated cerebral blood flow 45 min post dose, no changes related to placebo were seen on cognitive function or mood (Wightman et al., 2012). Another study showed that an acute (2 h) EGCG treatment was associated with increased calmness, reduced stress and increased EEG activity in the midline frontal and central brain regions (Scholey et al., 2012). However, these authors did not assess cognitive function. Despite these promising findings of acute doses on brain function and hemodynamic factors, clinical studies exploring the long-term effects of EGCG on cognitive function are required.

### Berberine and caffeine

Alkaloids, such as caffeine and berberine, the latter obtained from several plants including *Tinospora cordifolia*, have protective roles on the BBB. In rabbits with high cholesterol, caffeine consumption attenuated leakage of the inflammatory marker immunoglobulin G and Evans blue (used to measure the permeability of the BBB to macronutrients) to the brain tissue, indicating a possible role in protecting the BBB integrity (Chen et al., 2008). Caffeine is found in various beverages (e.g., coffee, tea) and food sources (e.g., chocolate) and is commonly consumed in our society. Caffeine suppresses the production of inflammatory cytokines (e.g., TNF-α, IL-10) and is thought to have protective effects on the BBB (see Horrigan et al., 2006; Chen et al., 2010 for review). The mechanism by which caffeine protects the BBB leakage is thought to be via inhibiting neuroinflammation as seen in *in vitro* models lacking a BBB and through modulating astrocytes, microglia and neurones (Chen et al., 2010).

## Plant based extracts

### Ginseng–*Panax quinquefolius*

Clinical studies have shown that the standardized ginseng (*Panax quinquefolius*) root extract, demonstrated improved cognitive function in healthy older individuals, particularly in working memory, spatial working memory and executive functioning domains (Ossoukhova et al., 2015). Ginseng's constituents' ginsenodies Rh2, Rh3, and compound K have anti-inflammatory effects through their ability to inhibit nitric oxide synthase and cytokine expression, and by stemming microglia-mediating mechanisms (Choi et al., 2011). Ginsenoside Rb1 in particular, protects against BBB dysfunction in subarachnoid hemorrhage brain injury rat models (Li et al., 2010). The reduction of neurological deficits, brain oedema and BBB permeability are argued to underlie BBB protection. Further to this Ke et al. (2014) demonstrated that ginsenoside Rb1 attenuates damage to rat cerebral cortical neurons against hypoxia-activated microglia. This neuroprotective mechanism may be mediated by the downregulation of nitric oxide, superoxide, and TNF-α expression (Ke et al., 2014). Ginseng's other mechanisms for improving cognitive function involve inhibiting microglial pathways, attenuating neuroinflammation (TNF-α and IL-6) and increasing acetylcholinesterase levels in the cortex and hippocampus (Xu et al., 2014). Furthermore, in an acute hippocampal injury model, resulting in spatial memory, learning and memory impairments, Xu and colleagues assessed the effects of ginsenosides (Rb_1_, Rb_3_ and Rd, termed Rb extract) on neuronal loss in rats. They found that Rb delayed microglial activation, prevented memory impairments, and protected astrocytes and neurones (Xu et al., 2014). In another study by the same research group, ginsenoside Rg1 treatment in aging rat models resulted in an attenuation of age associated changes in the hippocampus, including cognitive impairments and hippocampal neurogenesis compared with the controls (Zhu et al., 2014). Additionally, ginsenoside Rg1 treatment elevated age-associated biomarkers in the hippocampus including antioxidants (glutathione peroxidase and superoxide dismutase, decreased proinflammatory cytokine levels (IL-1b, IL-6, and TNF-α). Importantly, ginsenoside Rg1 treatment attenuated astrocyte activation, which may have been due to the anti-inflammatory and neurogenesis ability of this treatment (Zhu et al., 2014).

### Ginkgo biloba

*Ginkgo biloba* is a well-known plant based extract for its benefits on cognitive functioning through its antioxidant and vascular functions. Recent *in vitro* evidence suggests that an additional mechanism for cognitive enhancing effects of Ginkgolide B may be in reducing BBB permeability as shown in rats (Sharma et al., 2000). Recently Wan et al. (2014) discovered *in vitro*, that the well characterized *Ginkgo biloba* leave extract EGB-761, prevented brain endothelial damage caused by beta-amyloid oligomer, which plays a key role in the pathogenesis of AD (Wan et al., 2014). In human studies a *Ginkgo biloba* leaf extract decreased IL-6 serum levels in patients with neurologic disorders (Ching-Hsiang et al., 2012). A recent randomized controlled pilot study showed that following 1 week treatment with a special combination of *Panax ginseng, Ginkgo biloba*, and *Crocus sativus* (Sailuotong; SLT) improved working memory performance in healthy adults (Steiner et al., 2015).

### Bacopa monnieri

Clinical studies have reported in healthy subjects that compared to placebo, chronic *Bacopa monnieri* daily treatment (3 months; 300/320 mg/day, 150/160 mg × 2/day) improves visual information processing, learning rate, memory consolidation, anxiety levels, working memory, spatial working memory, attention, verbal learning, and cognitive processing (Stough et al., 2001, 2008; Peth-Nui et al., 2012). Additionally in AD patients, a higher dose of *Bacopa monnieri* (300 mg × 2/day) improved attention, language and comprehension following a 6 month intervention (Goswami et al., 2011). One mechanism by which *Bacopa monnieri* improves cognitive function may be through its ability to reduce inflammation. *Bacopa monnieri* inhibits cyclooxygenase (COX), down regulates TNF-α, inhibits ROS and reduces DNA damage in rat astrocytes, demonstrating its anti-inflammatory actions (Russo et al., 2003; Viji and Helen, 2008).

### Scutellaria baicalensis, Scutellaria laterifolia

Skullcap (*Scutellaria baicalensis*) is a herb traditionally used for relieving anxiety and stress. The root extract of the herb *Scutellaria baicalensis*, has been demonstrated *in vivo* studies to attenuate the BBB disruption through anti-inflammatory effects (the root extract *Scutellaria radis*) (Shin et al., 2012) and reduces BBB permeability (Zhu et al., 2012). Preclinical studies showed improvements in cognition in aged and senescent rat models (Song et al., 2009; Jeong et al., 2011). Additionally, reduced oxidative stress (MDA concentrations), increased antioxidant activity (superoxide dismutase, catalase) and reduced expression of inflammatory markers (iNOS, COX) were observed in aged rat brain tissue including the hippocampus and cerebral cortex following treatment with a special *Scutellaria baicalensis* extract (Song et al., 2009; Jeong et al., 2011). However, the effects on cognitive function in humans are mixed (Brock et al., 2014) and future clinical studies employing comprehensive neuropsychological test batteries are needed. Taken together these findings suggest that *Scutellaria baicalensis* may be a plant based extract to consider when researching conditions that involves the disruption of the BBB, elevated oxidative stress and reduced antioxidant activity, such as in cognitive impairments.

### Salvia triloba, Salvia officinalis

*Salvia miltiorrhiza* and *Salvia triloba* have been studied for their neuroprotective effects. *Salvia miltiorrhiza* commonly known, as Danshen is an herb used in Traditional Chinese Medicine. The terpine Tashinone IIA is one of *Salvia miltiorrhiza's* major active constitutes shown to maintain the integrity of the BBB and endothelial cell function (Wang et al., 2010; Zhang et al., 2010). Additionally, AD model rats treated with *Salvia triloba* and *Piper nigrum* showed significantly increased brain ACh levels, reduced brain and serum inflammatory marker levels (CRP, NF-jB_65_ and MCP-1 levels). Although few clinical studies have been conducted on Salvia, one randomized controlled trial showed that relative to placebo, 16 week administration of *Salvia officinalis*, produced significant improvements on the Alzheimer's Disease Assessment Scale cognitive subscale (ADAS-cog) scores in patients with mild to moderate dementia (Akhondzadeh et al., 2003). By ameliorating cholinergic dysfunction, reducing inflammation and increasing antioxidant activity, these plant based extracts may assist in repairing neuronal damage associated with AD (Ahmed et al., 2013a).

### Withania somnifera

Withanolides and extracts from *Withania somnifera* have been studied for their anti-inflammatory and immunomodulatory properties as well as their cognitive enhancing effects (Pingali et al., 2014; Gupta and Kaur, 2016). A recent *in vitro* study demonstrated that a leaf extract from *Withania somnifera* decreased production of the pro-inflammatory mediators TNF-α, IL1-β, IL6 as well as ROS via downregulation of NFκB proteins in inflamed primary microglial cells. Furthermore, the extract inhibited microglial migration, an important aspect of neuroinflammation, and induced apoptosis of the inflamed microglial cells (Gupta and Kaur, 2016). This study also suggests that *Withania somnifera* is capable of maintaining or restoring BBB integrity by inhibiting expression of microglial inflammatory factors as matrix metalloproteinases, associated with opening of the BBB (Rosenberg, 2002; Shigemori et al., 2006; Gupta and Kaur, 2016). In an *in-vitro* study a *Withania somnifera* leaf extract and the active compound withanone, but not withaferin A, showed to be protective against oxidative stress in brain-derived cells (Shah et al., 2015). A rat model showed that pre-treatment with *Withania somnifera* reversed the induced oxidative stress and the resulting cognitive decline via a strong antioxidant effect (Ahmed et al., 2013b).

## Other compounds

There are many other plant based and nutraceutical compounds that may be important therapeutic targets for cognitive aging via their actions on the immune system. These include Ginger, Vitamin D, Alpha lipoic acid, Omega-3 essential fatty acids, and Obovatol from *Magnolia obovata*. However, the mechanisms and or trials assessing cognition are lacking. As such we believe that it is too early to include these compounds in this review.

## Conclusion

Mechanisms including BBB integrity, oxidative stress, chronic stress, hippocampal neurogenesis, microglial activation and chronic low-grade neuroinflammation, have shown to be related to cognitive changes across age. Supplementation with one or more plant based extracts or nutraceuticals that act on these mechanisms may be an important next step toward preventing age associated cognitive decline. In recent years there has been a growing interest in exploring naturally forming compounds on protecting BBB permeability, ameliorating microglial activation and/or neuroinflammation, with the aim to develop treatments to prevent cognitive decline. Various plant based extracts have shown to exert protective effects on the BBB by preserving BBB integrity and function. These important compounds are found in plants (e.g., *Ginkgo biloba, Panax ginseng, and Bacopa monnieri*) and food sources (e.g., resveratrol, tea polyphenols, plant alkaloids and antioxidants). Since microglial cells contribute to neurodegenerative diseases by activating neuroinflammatory processes and oxidative stress, natural compounds that supress these mechanisms may be key therapies in preventing cognitive decline in older individuals. Potential antioxidant therapies are phytochemicals including curcumin, EGCG and resveratrol which interestingly also play a role on microglial cells.

However, the translational gap between *in vitro, in vivo* and clinical studies is still a major issue and there is a paucity of studies looking at the immunomodulatory effects of the discussed plant extracts and secondary plant metabolites in a healthy population. Moreover, careful consideration should be made in respect to the immunosuppressive and immunomodulatory effects of the plant extracts and active plant compounds, as for instance hypo-activity of microglial cells has likewise demonstrated to be involved in disturbing normal brain functioning (Niraula et al., 2017). Therefore, the long-term effects of the herbal treatments should be studied more extensively.

Another aspect to take into consideration is that humans present a broad range of responses to similar plant based extracts related to genetic and epigenetic modulations involved in the metabolism and distribution of the active compounds (Szarc vel Szic et al., 2015). Interestingly, modern science is investigating the effects of many traditionally used medicinal plant extracts and plant compounds and should perhaps also evaluate the great potential of other fundamental principles underlying traditional medicinal systems.

One fundamental principle in Ayurveda, the Indian traditional medicinal system, is the *prakriti*, which defines a person's true nature based on psychosomatic features (Prasher et al., 2017). The *prakriti* is independent of racial, ethnic and geographical factors and is highly correlated with molecular and genetic profiles (Joshi et al., 2010; Ghodke et al., 2011; Prasher et al., 2016). Therefore, future research could be focused on personalized herbal supplementation to prevent age-related cognitive decline and thus aim for an optimal response through a personalized rebalance of the various underlying mechanisms. Furthermore, some of the common cofounding factors that currently debilitate comparisons within and between research could be rectified. In addition, the *prakriti* is likely also underlying the discrepancies found between other study outcomes as either increased or decreased amounts of pro-inflammatory cytokines in similar research settings, because the homeostatic imbalance can take a different course depending on someone's *prakriti* (Prasher et al., 2016).

## Author contributions

CS conceptualized the review; All authors contributed to the writing of the review; CK took overall responsibility with the integration of the review.

### Conflict of interest statement

The authors declare that the research was conducted in the absence of any commercial or financial relationships that could be construed as a potential conflict of interest. Industry (Blackmores Australia) provided seed funding to write this review. No constraints were placed on the authors in terms of topics covered, material written, or publication.
